# Aerobic and Combined Exercise Sessions Reduce Glucose Variability in Type 2 Diabetes: Crossover Randomized Trial

**DOI:** 10.1371/journal.pone.0057733

**Published:** 2013-03-11

**Authors:** Franciele R. Figueira, Daniel Umpierre, Karina R. Casali, Pedro S. Tetelbom, Nicoli T. Henn, Jorge P. Ribeiro, Beatriz D. Schaan

**Affiliations:** 1 Exercise Pathophysiology Research Laboratory, Hospital de Clínicas de Porto Alegre, Porto Alegre, Rio Grande do Sul, Brazil; 2 Postgraduate Program in Endocrinology, Universidade Federal do Rio Grande do Sul,Porto Alegre, Rio Grande do Sul, Brazil; 3 Postgraduate Program in Cardiology, Universidade Federal do Rio Grande do Sul, Porto Alegre, Rio Grande do Sul, Brazil; 4 Instituto de Cardiologia/Fundação Universitária de Cardiologia do Rio Grande do Sul, Porto Alegre, Rio Grande do Sul, Brazil; 5 Department of Internal Medicine, Universidade Federal do Rio Grande do Sul, Porto Alegre, Rio Grande do Sul, Brazil; Brigham & Women’s Hospital, and Harvard Medical School, United States of America

## Abstract

**Purpose:**

To evaluate the effects of aerobic (AER) or aerobic plus resistance exercise (COMB) sessions on glucose levels and glucose variability in patients with type 2 diabetes. Additionally, we assessed conventional and non-conventional methods to analyze glucose variability derived from multiple measurements performed with continuous glucose monitoring system (CGMS).

**Methods:**

Fourteen patients with type 2 diabetes (56±2 years) wore a CGMS during 3 days. Participants randomly performed AER and COMB sessions, both in the morning (24 h after CGMS placement), and at least 7 days apart. Glucose variability was evaluated by glucose standard deviation, glucose variance, mean amplitude of glycemic excursions (MAGE), and glucose coefficient of variation (conventional methods) as well as by spectral and symbolic analysis (non-conventional methods).

**Results:**

Baseline fasting glycemia was 139±05 mg/dL and HbA1c 7.9±0.7%. Glucose levels decreased immediately after AER and COMB protocols by ∼16%, which was sustained for approximately 3 hours. Comparing the two exercise modalities, responses over a 24-h period after the sessions were similar for glucose levels, glucose variance and glucose coefficient of variation. In the symbolic analysis, increases in 0 V pattern (COMB, 67.0±7.1 *vs.* 76.0±6.3, P = 0.003) and decreases in 1 V pattern (COMB, 29.1±5.3 *vs.* 21.5±5.1, P = 0.004) were observed only after the COMB session.

**Conclusions:**

Both AER and COMB exercise modalities reduce glucose levels similarly for a short period of time. The use of non-conventional analysis indicates reduction of glucose variability after a single session of combined exercises.

**Trial Registration:**

Aerobic training, aerobic-resistance training and glucose profile (CGMS) in type 2 diabetes (CGMS exercise). ClinicalTrials.gov ID: NCT00887094.

## Introduction

The main objective in treating diabetes is to reduce HbA1c, as it reflects average glycemia over several months, and has strong predictive value for diabetes complications [1], [2]. Other components of dysglycemia, such as postprandial glucose and glucose variability, however, have been recently evaluated as possible targets for intervention. Glucose variability may contribute to the generation of excessive protein glycation and oxidative stress, which are key factors in the pathogenesis of diabetic complications [3]. Moreover, high glucose variability was shown to be associated with reduced endothelial function in patients with type 2 diabetes and optimal metabolic control [4]. However, the mean amplitude of glycemic excursions (MAGE) could not predict the development of retinopathy or nephropathy in a cohort of type 1 diabetic patients [5]. It is reasonable to speculate that the controversial findings regarding the association of glucose variability with outcomes in patients with diabetes [3]–[6] may result from limited tools to identify genuine disturbances in glucose variability.

Glucose curve, like any biological signal, has linear and non-linear properties that can be analyzed by statistical methods. The oscillatory nature of this signal seems to depend on physiological mechanisms related to free radicals [7], insulin sensitivity [8], and inflammatory markers [9] that modulate its behaviour. Accordingly, the application of spectral analysis could indicate the presence of senoidal components, allowing a quantitative and differentiated assessment.

Since age and disease are associated with loss of complexity in many physiological systems, complexity analysis techniques may provide information on these phenomena [10], [11]. As recently reported [12], the progressive loss of complexity in glycemic profile from health through metabolic syndrome to type 2 diabetes seems to precede hyperglycemia and correlates with other markers of disease progression, suggesting that non-linear analysis may be useful in the evaluation of the progression of dysglycemia to overt diabetes or related complications. Complexity analysis can be performed by several methods, including the evaluation of entropy and entropy rate through symbolic analysis [13].

Pharmacological agents acting on postprandial glucose excursions may attenuate glucose instability. Although lifestyle interventions have an important role in the treatment of type 2 diabetes [14], [15], [16], little is known about the effect of exercise in glucose variability, especially regarding whether different types of exercise modalities could have distinct impact on glucose variability. In the present study we aimed to assess the effect of a single exercise session on glucose levels and glucose variability in patients with type 2 diabetes, as evaluated by continuous glucose monitoring system (CGMS). To determine whether the post-exercise glucose levels would be influenced by different exercise modalities, we used two randomized sessions consisting of either aerobic (AER) or aerobic combined with resistance exercise (COMB). In order to separate the effects of meals and exercise sessions, the analysis was performed on data derived from all glucose curves, before and after exercise, divided in before and after meal, according to diary appointments. Glucose variability was evaluated by conventional methods (glucose standard deviation, glucose variance, MAGE and glucose coefficient of variation) and by non-conventional methods (spectral and symbolic analysis). As spectral and symbolic analyses had never been used in the evaluation of glucose variability, mathematic tools were applied for full validation of these methods for this signal.

## Methods

The protocol for this trial and supporting CONSORT checklist are available as supporting information; see Checklist S1 and Protocol S1.

### Research Design and Participants

**Figure 1:**
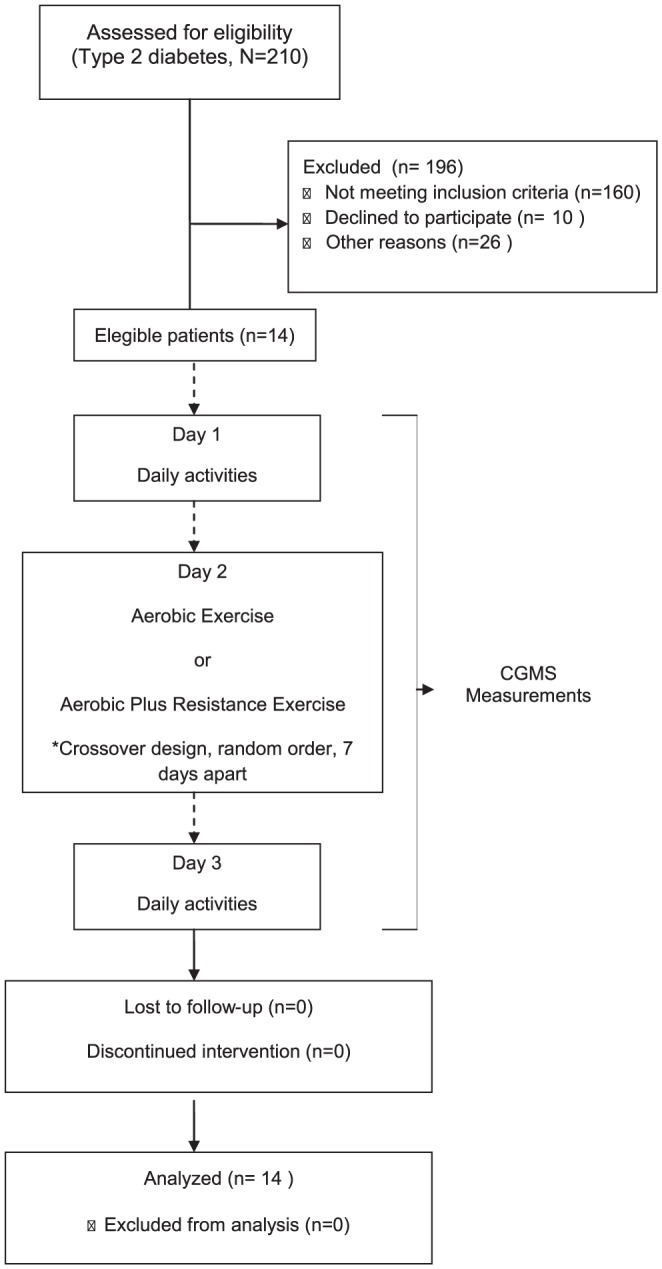
Flow Diagram.

Fourteen patients with type 2 diabetes participated in the experiments using a crossover randomized design (Figure 1). Individuals were recruited from outpatient clinics, and were not taking insulin or sulphonylureas. Exclusion criteria were heart disease, proliferative diabetic retinopathy, severe autonomic neuropathy, any limb amputation, uncontrolled hypertension, diabetic nephropathy (albuminuria>than 17 mg/L) and/or chronic renal failure (creatinine >1.4 mg/dL), and osteomuscular conditions which could limit the proposed protocols. The protocol was approved by the Ethics in Research Committee at Hospital de Clínicas de Porto Alegre and all patients provided their written informed consent before the participation. At the entry of the study, clinical characteristics, usual physical activity (International Physical Activity Questionnaire - IPAQ) [17], anthropometric evaluation, as well as 12-h fasting blood (glucose, HbA1c, cholesterol, HDL-cholesterol, triglycerides, creatinine) and urine (microalbuminuria) exams were obtained for each subject**.** One week prior to the experimental exercise session, subjects underwent maximal cardiopulmonary exercise testing and maximal strength testing.

### Maximal Cardiopulmonary Exercise Testing

The maximal incremental exercise test was performed on an electrically braked cycle ergometer (ER-900, Jaeger, Würzburg, Germany) with increments of 20 W/minute, as previously described [18]. During the test, gas exchange variables were measured by a previously validated system (Oxycon Delta, VIASYS, Healthcare GmbH, Jaeger, Germany). Heart rate continuously monitored by a 12-lead electrocardiogram (Nihon Kohden Corporation, Japan) and blood pressure was measured with automatic oscillometric device every 2 min.

### Strength Testing

Strength was measured by 1 repetition maximum (1-RM), which was preceded by exercises at mild intensity for movement familiarization and warm-up. Proper technique was demonstrated and practiced for leg press exercise (Sculptor, Porto Alegre Brazil), leg extension, bench press and biceps curl. When new attempts were needed, a 5-min resting period was allowed between subsequent attempts.

### CGMS Measurements

Subjects were admitted to the laboratory in the morning at approximately 9∶00 a.m., 24 h before the exercise session, when the glucose sensor (Sof-Sensor™, Medtronic Mini-Med, Northridge, USA) was inserted subcutaneously. The sensor is a glucose oxidase based platinum electrode that is inserted through a needle into the subcutaneous tissue of the anterior abdominal wall, using a spring-loaded device (Senserter, Medtronic, Northridge, USA). Glucose oxidase catalyzes the oxidation of glucose in the interstitial fluid, which generates an electrical current. The current is carried by a cable to a pager-size monitor that analyzes the data every 10 s and reports average values every 5 min, totalizing 288 readings per day. Glucose profiles were collected the day before (day 1), the day of (day 2), and the day following (day 3) the single 1-h bout of moderate exercise. Sensor readings were calibrated with a glucose monitor (Accu-Check Performa, Roche Diagnostics, Mannheim, Germany) using 4 finger-stick blood samples for each 24 h. Each sensor was used continuously for up to 72 h. All patients were previously instructed about the operation of the monitor, which included event registration for meals, inserting capillary glucose values as calibration, general care and completing detailed food recall during the period of monitoring. Subjects were asked to closely match their daily nutritional intake, avoid physical exercise except for the protocol, and record detailed food intake and daily physical activities in diaries across the 3-d period. The dietary assessment was performed using the software DietWin (Porto Alegre, Brazil).

### Glucose Variability Evaluation

**Figure 2:**
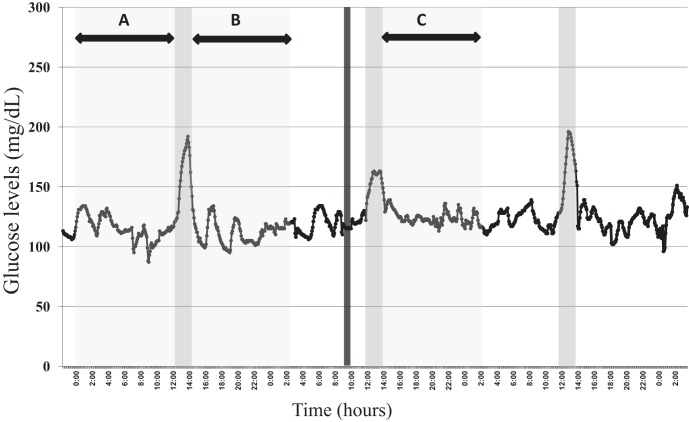
Example of a glucose curve obtained from CGMS record of 72 hours depicting the time periods used to evaluate the effects of meals, pre (section A) and post prandial (section B), and exercise, pre (section B) and post (section C) sessions, indicated in blocks. The dark grey line represents the period of exercise session.

Glucose variability was assessed from series of absolute values of glucose, obtained by CGMS, sampled every 5 minutes. Each method was used as reported in the literature and respecting its limitations. Glucose variability was evaluated using conventional analysis and other mathematical methods generally applied to biological series, here called non-conventional analysis of glucose variability. The selection of the segments for the following analysis is illustrated in Figure 2.

Conventional analysis of glucose variability was constructed from the statistical properties of the series, obtaining the following indices: MAGE [19], glucose variance (VAR), glucose coefficient of variation (CV%), and glucose standard deviation (SD), all normalized by the mean blood glucose in each period [20]–[23]. These indices, except MAGE, were calculated for every 6-h block to obtain the measures according to the period of the day. The MAGE index was calculated for the whole signal (24 h), and its calculation is based on the differences between consecutive points, considering those which are higher than one standard deviation [19]. In this way, according to the characteristics of the curves in our study, the MAGE index was calculated only for the highest curve between 12 h and 14 h.

Non-conventional analyses were applied in the pre- and the postprandial periods before the exercise session, and in the postprandial period after the exercise sessions. The first set period for the analysis was the one obtained before exercise, which was compared to the one obtained after the AER and COMB exercise sessions. Non-conventional analysis of glucose variability was conducted using two methods applied to the glucose series: a linear method based on spectral analysis and an integrated nonlinear approach to the complexity analysis, symbolic analysis. Spectral analysis is a linear method that allows quantifying the oscillatory components from time series, by autoregressive model, widely applied to heart rate and arterial pressure series [24]. A peak detector algorithm was used to determine possible predominant bands in the frequency power spectrum. The same methodology was applied to 10 surrogate signals, generated from each segment, to test false spectral results [25].

Symbolic dynamics relies on the calculation of Shannon entropy (SE) of the distribution of patterns lasting three measures and the classification of frequent deterministic patterns lasting three measures. Entropy calculates the degree of complexity of the distribution of the samples of a signal. The largest entropy is obtained when the distribution is flat (the samples are identically distributed). In contrast, the entropy decreases whenever values are distributed closer to a Gaussian sample distribution. Usually, in the analysis of biological variability, entropy is not calculated directly over the samples of the series but over patterns of length L (i.e., ordered sequences of (L-1) samples). Differently, entropy rate (e.g., approximated entropy or conditional entropy) provides a global index of complexity of the distributions of the samples conditioned to (L-1) previous samples as a function of L [26]. Symbolic analysis is an approach based on quantification of complexity that allows an advanced characterization of glucose variability series and the identification of experimental conditions known to differently perturb glucose oscillations. This method was fully described and validated previously [26]. Each subject and each experimental condition had its own range of glucose variations. Therefore, the full range of the sequences was uniformly spread on 6 levels (from 0 to 5), and presence of patterns was quantified. A redundancy reduction criterion was proposed to distribute deterministic patterns of the group in four categories according to the number and type of glucose changes: 1) no variation (0 V); 2) one variation (1 V); and 3) two like variations (2 LV); 4) two unlike variations (2 UV). The Shannon entropy and corrected conditional entropy (CCE) of the distribution of the patterns were calculated to provide a quantification of the complexity of the pattern distribution [26]. Probability density function (PDF) provides the distribution of the patterns and reports the sample frequency of each pattern as a function of the decimal code [26]. The small number of quantization levels utilized to describe this dynamics can express some patterns more frequent than others only by chance. In order to rule out this possibility, 20 surrogate data series were obtained from each original series and the results were statistically compared.

The surrogate signals were generated by routines implemented in Matlab, based on the works of Theiler et al. (1992) [27]. The same algorithm is used to generate surrogates in previous workw by our group [28], [29].Twenty surrogate data series were obtained from the original one by randomly shuffling the samples according to 20 different white noise realizations, thus completely destroying the original power spectrum but maintaining sample distribution. A complexity index (SE **or** MP%) calculated over the original series (CIO) was compared with those obtained from the surrogate series (CIS) by using the formula:
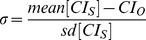



Where mean and sd represented the mean and the standard deviation over the 20 surrogate series. If σ >2, CIO could not be derived from a white noise with the same sample distribution as the original series (p<0.05) [27].

### Hiperglycemic Episodes Evaluation

To quantify the number of hyperglycemic episodes 24 h before and after the exercise we used the number of CGMS measurements above 150 mg/dL normalized by the total number of CGMS measurements during 24 h.

### Randomization

Subjects participated in the experimental sessions according to a computer-generated randomization. Such process occurred once before subjects were recruited in the study, indicating the order of the two 50-min sessions of either aerobic exercise (AER, n = 14) or combined aerobic and resistance exercise (COMB, n = 14), which were separated by at least 7 days. A researcher who was not part of the interventions handled the randomization.

### Exercise Protocol

Two exercise sessions, consisting of either AER or COMB exercise protocols, were carried out in a randomized order. Exercise intensity was recorded for each individual by a heart rate monitor (Polar F1 TM, Polar Electro Oy, Helsinki, Finland), and a Borg 0–10 scale was used to register individuals’ perceived exertion every 5 minutes throughout the experimental sessions. For the AER session, subjects exercised on a cycle ergometer (Embreex 360, Brusque, Brazil**)**. Each session included a 5-min warm-up at 20 watts, followed by 40 min at 70% of the peak heart rate, as determined in the incremental exercise test, and 5-min of stretching exercise as cool down. In the COMB session, subjects completed a 5-min warm-up on the cycle ergometer and continued for 20 min at 70% of the peak heart rate. This aerobic part was then complemented by 4 resistance exercises (leg press, leg extension, bench press and biceps curl), in which subjects completed 3 sets of 12 repetitions at 65% of 1-RM.

### Statistical Analysis

The 18.0 version of Statistical Package for Social Sciences software was used for statistical analysis. Descriptive data are presented as mean and SEM or median and interquartile range (P25–P75). Logarithmic transformation was applied to non normal distribution data. Glucose area under curve (AUC) value was calculated considering the integrated over area enclosed by the glucose curve, on each 24-h time interval. The effects of the interventions were compared by two-way analysis of variance for repeated measures (ANOVA), and multiple comparisons were performed with the Bonferroni correction.

## Results

Between March 2009 and December 2011, 210 patients with type 2 diabetes were screened for participation but only 14 participated in the protocols. Demographic and clinical characteristics of participants are shown in Table 1. Patients were 56±2 years old, predominantly women, had diabetes for 4.5 years (3.1–5.9), and did not present metabolic decompensation. Most of the patients were insufficiently active and this was reflected on reduced values of peak oxygen uptake. Dietary records were similar among the three days of the study with AER session (P = 0.56) and with COMB session (P = 0.43). No differences in macronutrient intake were observed when the three days including the AER session were compared with the three days including the COMB session (P = 0.79). On average, the subjects consumed their meals 156±78 min before and 97±42 min after the AER session, whereas meal intake was 147±69 min before and 117±87 min after the COMB session, respectively.

**Table 1:** Baseline characteristics of the patients studied.

Characteristics	
Men (n and %)	5 (35.7)
Age (yr)	56±2
**Anthropometrics**	
Body weight (kg)	79±4
Body mass index (kg.m^−2^)	30±1
Waist circumference (cm)	101±3
**Blood pressure (mmHg)**	
Systolic	136±4
Diastolic	77±2
Duration of diabetes (yr)	4.5 (3.1–5.9)
HbAlc (%)	7.9±0.7
Fasting blood glucose (mg/dl)	139±5
Total cholesterol (mg/dl)	191±8
HDL cholesterol (mg/dl)	61±13
LDL cholesterol (mg/dl)	95±12
Triglycerides (mg/dl)	161 (124–198)
**Psysical activity level (IPAQ)**	
Insufficiently active	11 (71.4)
Sufficiently active	3 (21.4)
Very active	1 (7.1)
VO_2_ peak (mL.Kg^−1^.min^−1^)	23±2
Heart rate peak (bpm)	156±6
R_peak_	1.2±0.0
**Maximal strength testing (1-RM) Kg**	
Biceps curl	9±1
Bench press	30±2
Leg press	131±17
Leg extension	35±3
**Medications (n and %)**	
Metformin	14 (100)
β-blockers	2 (14.3)
Angiotensin-converting enzyme inhibitors	4 (28.6)
Aspirin	4 (28.6)
Diuretics	6 (42.8)
Statins	5 (35.7)

HbA1c: glycated hemoglobin; VO_2_peak: peak oxygen uptake per kilogram of body weight/fat-free mass; R_peak_ peak respiratory exchange ratio of peak.

** Data are expressed as mean ± SEM, except duration of diabetes, which is expressed as median (95% CI); categorical variables are presented as numbers (%).

Table 2 shows the effects of a meal on glucose variability evaluated by conventional and non-conventional analyses. The MAGE index could not be evaluated before meal because its value depends on postprandial oscillations. For all other variables analyzed, the coefficient of variation was the only that was changed by the meal, showing a reduction of glucose variability after it (P = 0.008).

**Table 2:** Glucose variability evaluated by conventional and non-conventional analysis before and after meals, before any exercise session.

	Pre-prandial	Post-prandial
**Conventional**		
Glucose variance (mg^2^/dL^2^)	421.4±795.0	524.2±768.8
Coefficient of variation (%)	11.7±6.0	8.4±4.0*
Glucose standard deviation (mg/dL)	16.4±12.5	20.0±11.4
MAGE	-	42.4±119.8
**Non-conventional**		
*Spectral analysis*		
Total power (mg^2^/dL^2^)	92.8±26.2	135.2±33.5
Predominant band (peak at 0.002 Hz) (mg^2^/dL^2^)	4.4±1.1	9.8±5.0
*Symbolic analysis*		
Shannon entropy	4.2±0.05	4.2±0.08
0 V pattern (%)	54.2±2.0	48.4±2.6
1 V pattern (%)	37.7±1.4	41.4±2.0
2 LV pattern (%)	5.0±0.9	5.5±1.2
2 UV pattern (%)	3.1±0.6	4.6±0.8

Comparisons were performed by Student’s *t*-test;

* P<0.05 *vs.* pre-prandial. Data are expressed as mean ± SEM. MAGE index could not be evaluated before meal because its value depends on postprandial oscillations.

All participants completed AER and COMB exercise interventions, and levels of perceived exertion were similar during both experimental sessions (AER, 4.9±0.4; COMB, 4.8±0.5; P = 0.92). Glucose levels (CGMS), heart rate, systolic and diastolic blood pressure measured before and at the end of the exercise sessions are presented in Table 3. Both exercise modalities elicited similar increases in heart rate and blood pressure as well as similar reductions in glucose levels.

**Table 3:** Cardiovascular and metabolic responses to exercise.

	Aerobic		Combined		P	P	P
	Pre	Post	Pre	Post	Exercise	Time	Interaction
Heart rate (bpm)	86±2	113±2	84±3	109±8	0.29	0.001	0.58
Systolic blood pressure (mmHg)	130±5	145±7	129±4	134±5	0.20	0.005	0.29
Glucose levels evaluated by CGMS (mg/dl)	151±8	124±9	147±9	125±6	0.89	0.001	0.45

P: Two-way analysis of variance for repeated measures (ANOVA).Data are expressed as mean ± SEM.

**Figure 3:**
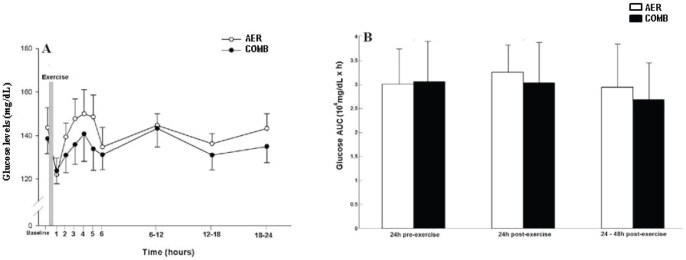
24-h glucose levels (CGMS), detailed on the first 6 **h after exercise in aerobic (AER) and aerobic plus resistance (COMB) exercise (Panel A) exercise sessions.** Panel B shows 24-h glucose levels (CGMS) as the area under the glucose response curve comparing AER and COMB sessions (P = 0.335). Data are reported as mean ± SEM.

Figure 3 (panel A) shows glucose levels determined by CGMS after AER and COMB exercise sessions. For both exercise modalities, glucose levels returned to baseline levels similarly about 3 hours after exercise; this reduction in glucose levels was not different comparing 3 hours after exercise *vs.* baseline (AER session P = 0.99; COMB session P = 1.00; α = 0.05 for Pre/Post P = 0.050). In addition, Figure 3 (panel B) shows that the AUC of glucose over 24 h after exercise was similar in both exercise protocols. During the day before the AER session, hyperglycemic episodes (expressed as the percentage of time glucose levels were above 150 mg/dL) occurred in 33±0.3% of the period, whereas on the day after the session, such episodes occurred in 31±0.3% of the 24 h period (P = 0.79). Before the COMB session, hyperglycemic episodes occurred in 31±0.2% of the readings throughout the 24-h period, whereas such episodes occurred in 27±0.3% of the readings during the 24 h after the COMB session (P = 0.33). The duration of hyperglycemic episodes was not different after both exercises (P = 0.50).

**Figure 4:**
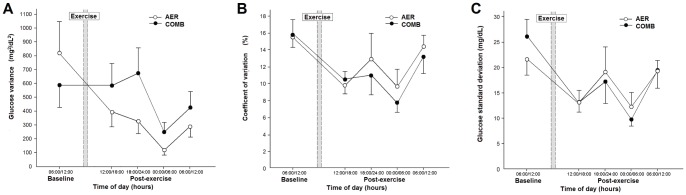
Glucose variability evaluated by conventional analysis before and after aerobic (AER) or aerobic plus resistance exercise (COMB) exercise. Panel A, glucose variance; Panel B, coefficient of variation and Panel C, standard deviation. Data are reported as mean ± SEM; n = 14. Two-way analysis of variance for repeated measures (ANOVA); Bonferroni correction.

Conventional analysis of glucose variability is shown in Figure 4. Panel A shows that glucose variability, as evaluated by glucose variance, was similar after exercise for AER and COMB sessions. Similar findings were obtained for glucose variability evaluated by the glucose coefficient of variation (Panel B) and glucose standard deviation (Panel C).

Non-conventional analysis of glucose variability (spectral analysis results) indicates the absence of a predominant band in surrogate series and the presence of a band centered at 0.002 Hz in all analyzed series. Similar spectrum parameters were presented after exercise for the two exercise modalities (AER, 7.3±8.7 *vs.* 3.1±2.3 mg^2^/dl^2^, P = 0.11; COMB, 5.9±8.4 *vs.* 3.8±5.6 mg^2^/dl^2^, P = 0.13).

**Figure 5:**
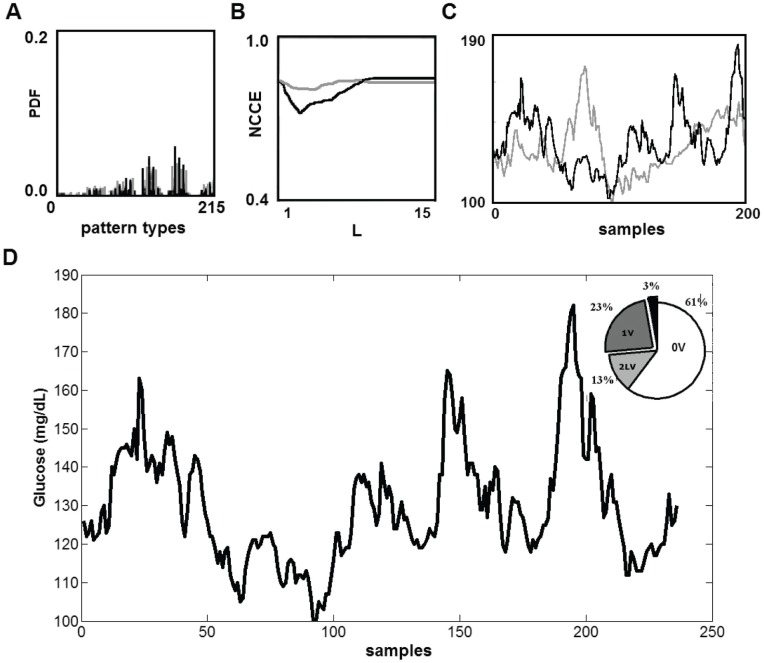
Glucose variability evaluated by non-conventional analysis: symbolic analysis and the mathematical analysis to method validation. Panel A: Comparison between original (black line) and surrogated (gray line) series with respect to Probability density function (PDF), indicating a random result in surrogate series. Panel B: Normalized corrected conditional entropy (NCCE), exhibiting a deep minimum in original series. Panel C: Example of an original and a surrogated series. Panel D: Example of symbolic analysis results. One way repeated measures ANOVA.

**Figure 6:**
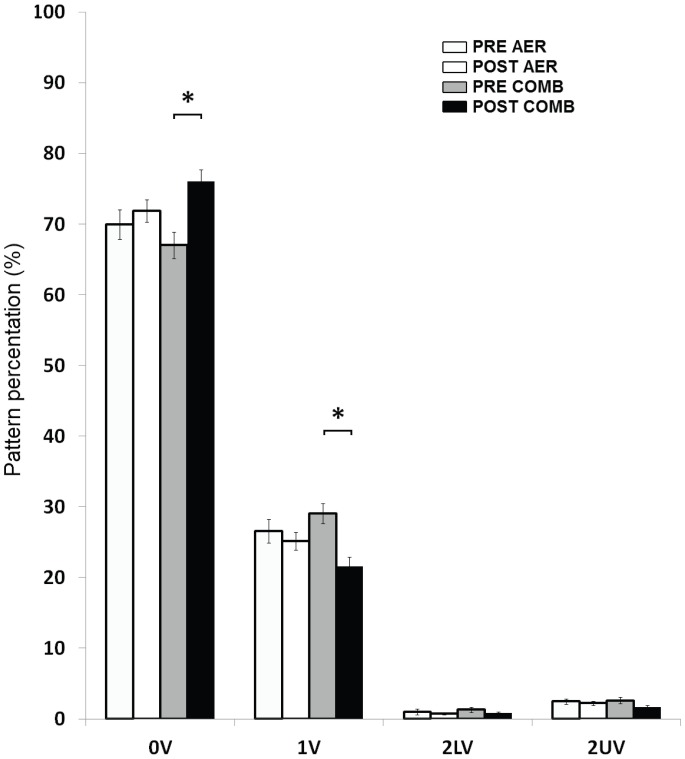
Results of glucose variability evaluated by symbolic analysis after AER or COMB exercise (n = 14) and comparison between before (open bars) and after (close bars) both exercises. Occurrence percentage of each pattern: 0 V (no variation), 1 V (one variation), 2 LV (two low variations) and 2 UV (two up variations). Data are reported as mean ± SEM. *P<0.01 *vs*. pre-exercise values (COMB), Two way repeated measures ANOVA.

Non-conventional analysis of glucose variability (symbolic analysis) is shown in Figures 5 and 6. Figure 5 depicts the mathematical analysis used for validation of the analysis applied. Probability density function obtained from original series was different from that obtained from its surrogate series, without a predominant pattern, indicating a random result (Panel A). Panel B depicts normalized corrected conditional entropy (NCCEs), calculated over the original (black line) and surrogate series (gray line). The NCCE from original series exhibited a deep minimum, while the surrogated data were completely flat. Panel C shows an example of original and surrogated series generated to method validation and Panel D illustrates an example of symbolic analysis results applied to real glucose series. Figure 6 shows the changes induced by exercise sessions in the patterns of symbolic analysis. Results obtained comparing exercise sessions reported differences between them on 0 V, which is related to no variation in glucose short-term variability (three consecutive measures), only for COMB sessions compared to pre-exercise values (AER, 69.9±7.8 *vs.* 71.9±7.1, P = 0.35; COMB, 67.0±7.1 *vs.* 76.0±6.3, P = 0.003, power of performed test (α = 0.05) = 0.927) and 1 V patterns (AE, 26.6±6.2 *vs.* 25.2±4.7, P = 0.47; COMB, 29.1±5.3 *vs.* 21.5±5.1, P = 0.004, power of performed test (α = 0.05) = 0.952). The Shannon entropy did not change after both exercise sessions (AER, 4.3±0.3 *vs.* 4.1±0.3, P = 0.19; COMB, 4.2±0.2 *vs.* 4.2±0.3, P = 0.52).

## Discussion

In the present study, we show that acute sessions of AER and COMB promote similar reductions in glucose levels as evaluated by the CGMS immediately after the exercise, which are sustained for approximately 4 hours after the exercise. Moreover, although absolute glucose level reduction was small, the pattern of glucose curve was changed, indicating a reduction in its variability as evaluated by non-conventional methods, which was undetectable when conventional methods were applied.

The acute glucose lowering effect of exercise results from high muscle glucose uptake determined by increased insulin sensitivity induced by muscle contraction [30], and this effect has been described to last up to 72 h after a single exercise bout [31]. In contrast, our data, using multiple and frequent glucose measurements with continuous monitoring, do not confirm such long duration of glucose reduction after either AER or COMB exercise sessions. The increased insulin sensitivity determined by exercise is accomplished by increased GLUT4 protein [32–30] which is determined by enhanced skeletal muscle AMPK activity [30]–[33]. These changes occur very rapidly after the exercise onset and can last 16 h after the intervention [32]. However, feeding a high-carbohydrate diet, with development of glycogen supercompensation, prevents the increase in insulin responsiveness [34], whereas feeding a carbohydrate-free diet maintains the increase in GLUT4 and insulin responsiveness for days after the exercise bout [35]. Considering that patients in our study maintained their usual carbohydrate ingestion, this could be one reason influencing the reduced duration of the exercise-induced hypoglycemic effect. Moreover, the severity of the insulin-resistant state characteristic of type 2 diabetes has been shown to preclude the expected exercise increase in glucose uptake by muscle cells [36].

Considering that sulphonylureas [37] and insulin [35]–[38] may increase the hypoglycemic action of exercise when the treatments are combined, we studied patients who were not using these agents, in order to isolate the effects of exercise. Because current guidelines recommend metformin as the first step in type 2 diabetes treatment, and this drug has no hypoglycemic effect, patients on metformin were included. However, recent data have shown that the enhanced insulin sensitivity caused by an exercise bout is blunted by the administration of metformin, which was accounted for the absence of skeletal muscle AMPK activity rise, thus resulting in an attenuation of the well-documented hypoglycemic effect of exercise alone [33]. This is another fact possibly contributing to small lowering of absolute glucose observed in the present study. Moreover, the effect of metformin in lowering plasma glucose after a meal has been shown to be attenuated by exercise [39].

To track changes in blood glucose levels of patients both in usual activities, as well as during exercises sessions, we used the CGMS, which has proven to be effective in registering changes in glucose levels in usual activities, recording nocturnal hypoglycemia and postprandial hyperglycemia that are not evident with routine monitoring [40]. Furthermore, in a previous study, we showed that in patients with diabetes, agreement between finger-stick blood glucose and CGMS-measured glucose during exercise was within the recommended accuracy limits considering the proposed International Organization for Standardization (ISO) [41].

Results from glucose obtained with the CGMS during an acute exercise session were previously reported by other authors [35]–[42], but, to our knowledge, the present study is the first to compare an acute session of aerobic exercise with a combination of aerobic with resistance exercise. The similar reduction in glucose levels after AER or COMB exercise sessions are in accordance with the findings of our recent meta-analysis on the chronic effects of exercise on HbA1c in patients with type 2 diabetes [16]. Since controversial results in long-term trials [14], [15] might be related to the amount of exercise performed, we have been careful to apply the same duration of both exercise protocols. In addition, a modified Borg CR10 scale was used to measure the rating of perceived exertion, indicating similar perceived exertion between the two exercise sessions.

An interesting new finding of the present study was the reduction in glucose variability induced by acute exercise sessions, irrespective of the modality employed. Glucose fluctuations trigger activation of oxidative stress, a main mechanism leading to diabetic complications [3]. However, different ways of evaluating glucose variability have provided controversial results concerning their impact to improve the risk prediction of microvascular complications above the risk predicted by HbA1c levels [5], [6]. Importantly, these studies were performed in patients with type 1 diabetes, and no study evaluated the possible beneficial effect of exercise training as a tool for lowering glucose variability and its potential in reducing diabetic complications in the long term. To our knowledge, the present study provides the first piece of evidence showing this potential by a single exercise session.

With regard to the results obtained with the non-conventional methods, we found some parameters were altered after the exercise sessions. The interpretation of these results requires attention to some important concepts related to the physiological mechanisms involved in the generation of glucose oscillations. First, the proposed validation using surrogated signals demonstrated the applicability of the method and the changes detected only in real signals, indicated the veracity of such characteristics present in the glucose curves, minimizing the possibility of intrinsic errors into the signal pattern. Second, spectral analysis indicated the presence of a slow physiological mechanism acting within a period of around 8 minutes (predominant band at around 0.002 Hz). The power of this band was not affected by meals, but was reduced after the exercise sessions. Moreover, the results of symbolic analysis were also not affected by meals, however, COMB exercise induced an increase of 0 V pattern, accompanied by reduction in the 1 V pattern. These changes, favoring 0 V pattern, represent no variation, indicating a decrease in glucose variability when evaluated on three consecutive measures. It is important to note that the results of non conventional methods can provide complementary information about glucose oscilation. Certainly, the most important information provided by our results is the detection of glucose curve features that were not previously detected by the indices conventionally used in the literature.

Interestingly, the question of complexity and pathology is not yet clear. Some studies have shown a relationship between pathological conditions and loss of complexity [43] but, on the other hand, but others suggest a possible opposite relationship [44]. Our study evaluates the acute effect of two sessions that evoke different physiological responses. Reducing complexity after the COMB sessions may not be related to an impaired systemic context, but to the predominance of a nonlinear mechanism or the withdrawal of a nonlinear mechanism shortly after the session. The mechanisms involved should still be studied to clarify the observed patterns of linearity.

We can only speculate on possible physiological mechanisms acting on the predominant frequency range of the power spectra and symbolic patterns described above. The responses observed in the present study may have been underlying the responses observed in the present stuy, there may have been hormonal actions linked to insulin synthesis and release [45], oxidative stress [46], and/or inflammatory factors [47]. Additionally, mechanisms directly affected by muscle contractions cannot be ruled out. The Akt substrate of 160 kDa (AS160), which is stimulated by insulin as well as by exercise, and its increase after exclusive sessions of either aerobic or resistance exercises may have contributed to change glucose dynamics [48], [49]. Moreover, since only the COMB session changed the glucose variability pattern, it is possible that additive effects from aerobic and resistance stimuli may affect the post-exercise insulin signaling. Some studies have shown a correlation between such parameters and glucose variability, both under physiological and pathological situations, indicating a possible influence of these mechanisms on the changed variables [50]–[54]. Therefore, further investigation is required in order to interpret the obtained results, since the isolated actions of these mechanisms on the parameters here addressed were not studied.

In conclusion, both AER and COMB exercise modalities in patients with type 2 diabetes using metformin were associated with small glucose level reductions, which lasted a short time. The glucose variability pattern was changed after COMB sessions only when non-conventional methods (symbolic analysis) were employed. Further research is needed to identify physiological mechanisms related to these mathematical characteristics. Long-term potential benefits of lowering glucose variability by exercise training should be tested in randomized clinical trials.

## Supporting Information

Checklist S1 PRISMA checklist.
(DOC)

Protocol S1 Trial protocol.
(DOC)
