# Supplementary material for: Aerobic and Combined Exercise Sessions Reduce Glucose Variability in Type 2 Diabetes: Crossover Randomized Trial
Source: PLoS One. 2013 Mar 11;8(3):e57733. doi: 10.1371/journal.pone.0057733 (PMC3594238; doi:10.1371/journal.pone.0057733)
Supplement: Protocol S1 — Trial protocol. (DOC) [file pone.0057733.s002.doc]

Universidade Federal do Rio Grande do Sul

Hospital de Clínicas de Porto Alegre

**Protocol S1: Trial Protocol**

**Efeitos Agudos e Subagudos do Exercício Físico Aeróbico e Aeróbico/Resistido em Pacientes com Diabetes Tipo 2 avaliados através do Sistema de Monitorização Contínua de Glicose**

**Franciele Ramos Figueira**

**Pesquisador responsável:** Profa. Dra. Beatriz D’Agord Schaan, Serviço de Endocrinologia, HCPA/UFRGS

**Co-Autores:**

Prof. Dr. Jorge Pinto Ribeiro, Serviço de Cardiologia, LaFiEx, HCPA/UFRGS

Dr. Daniel Umpierre, LaFiex, HCPA.

Dr. Karina R. Casali, LaFiex, HCPA

Pedro S. Tetelbom, LaFiex.

Nicoli T. Henn; LaFiex.

**Colaboração:**

Dr Ricardo Stein, Serviço de Cardiologia, LaFiEx, HCPA/UFRGS

Prof. Dr. Jorge Esteves, HCPA/UFRGS

Licia Pierosan, LaFiex.

Porto Alegre / 2008

## ÍNDICE

**Introdução 3**

**Objetivos 9**

**Hipóteses 10**

**Materiais e Métodos 11**

**Orçamento 20**

**Cronograma 21**

**Referência Bibliográfica 22**

**ANEXO I 25**

**ANEXO II 26**

**ANEXO III 28**

# 1 Introdução

O Diabetes Mellitus (DM) é uma doença crônica com elevado impacto individual e coletivo em termos de morbidade e mortalidade. No Brasil, dados nacionais de 2001 apontaram para uma prevalência na população acima de 40 anos de idade de 10% (1), enquanto que dados do RS mostram uma prevalência de 12,4% (2). A atividade física associada à dieta alimentar são medidas úteis na prevenção (3) e tratamento do DM tipo 2 (DMT2).

No DMT2, exercício físico regular melhora o controle glicêmico (4), reduz fatores de risco cardiovasculares e contribui para a perda de peso. Para melhorar o controle glicêmico a Associação Americana de Diabetes recomenda a prática de pelo menos 150 minutos de exercício aeróbico de intensidade moderada (40-60% da freqüência cardíaca máxima), distribuídos ao longo da semana, evitando mais do que 2 dias consecutivos sem atividade. Na ausência de contra-indicações, deve ser encorajada a prática de exercício de resistência 3 vezes por semana, nos principais grupos musculares (5).

Em indivíduos saudáveis, durante a realização do exercício físico ocorrem várias respostas fisiológicas cardiovasculares e metabólicas adaptativas. O consumo de oxigênio corporal total aumenta quase 20 vezes, o que é maior ainda na musculatura exercitada. A energia para este aumento de consumo provém de estoques musculares de glicogênio, triglicerídeos e ácidos graxos livres (AGL) e de glicose de origem hepática. Mesmo com este alto consumo muscular, a glicemia se mantém em níveis muito estáveis, devido à redução da insulinemia e aumento do glucagon plasmático. Cronicamente, além dessas adaptações hormonais, aumento de catecolaminas também é importante. A prática de exercícios físicos regulares traz uma série de benefícios, tais como redução do risco cardiovascular, de acidente vascular cerebral, do desenvolvimento de hipertensão arterial, DMT2, osteoporose, obesidade, câncer de cólon, câncer de mama, ansiedade e depressão (6).

Nos pacientes com DMT2 a atividade física regular melhora a sensibilidade à insulina prejudicada pela patologia de base, auxiliando na obtenção de melhor controle glicêmico, efeito não mediado pela perda de peso (7). O efeito de maior sensibilidade à insulina induzido por uma única sessão de exercício físico aeróbico pode durar entre 24 e 72h, dependendo da sua intensidade e duração (8), podendo ser maior para os exercícios resistidos (9).

Nos últimos 15 anos vêm aumentando as evidências de que a prática de exercícios resistidos deveria ser incluída nos programas de treinamento de indivíduos de todas as idades e com DMT2. Com a idade há uma tendência de redução da massa muscular, menor capacidade funcional, menor taxa de metabolismo basal, aumento de adiposidade e resistência à insulina, todas alterações passíveis de melhoria com os exercícios resistidos (10-12). Em pacientes com DMT2 dois estudos mostraram que um programa envolvendo múltiplos exercícios de alta intensidade 3 vezes por semana determina redução significativa da hemoglobina glicada (HbA1c), sem ser acompanhado de efeitos deletérios (13, 14). Recentemente estudo envolvendo 251 indivíduos com DMT2 comparou treinamento físico aeróbico e combinado aeróbico + resistido realizados pelos pacientes por até 6 meses, mostrando maior benefício quanto ao controle glicêmico com a segunda modalidade (15). É necessário considerar as contra-indicações para a prática dos exercícios resistidos, que incluem diabetes ou hipertensão arterial não controlados, neuropatia autonômica grave, neuropatia periférica grave e retinopatia proliferativa (16).

Embora tanto o treinamento aeróbico quanto o treinamento resistido tenham se mostrado benéficos para os portadores de DMT2 pouco se conhece sobre o perfil glicêmico durante, imediatamente após e em curto prazo após o exercício. A maioria dos estudos aborda o efeito crônico da atividade física regular, ou seja, aquelas adaptações produzidas pelo exercício físico ao longo do tempo. Porém, para que exista efeito benéfico ao longo do tempo, é preciso entender primeiro os efeitos agudos e subagudos do exercício físico, ou seja, aquelas adaptações que o organismo sofre durante a sessão de treinamento físico e imediatamente após o treinamento.

Na década de 80, um dos grandes avanços quanto ao controle glicêmico do diabetes foi a medida de glicemia capilar, realizada com amostras coletadas em ponta de dedo, hoje considerada indispensável para o tratamento do DM. No entanto a monitorização da glicemia capilar fornece apenas dados intermitentes, não permitindo visualização completa do perfil glicêmico. O Sistema de Monitorização Continua de Glicose (CGMS) consiste de um sensor instalado no tecido subcutâneo, pelo qual é possível registrar as glicemias ao longo do dia de maneira ininterrupta. Este método auxilia no monitoramento de pacientes, especialmente em condições clínicas que cursam com oscilações dos níveis de glicemia, permitindo que um profissional especializado possa readequar melhor a terapêutica idealizando melhor controle glicêmico (17). A medida da glicemia ocorre com base na reação eletroquímica da enzima glicose-oxidase encontrada no sensor, com a glicose do fluido intersticial. São identificados valores de 40 a 400mg/dl, captados a cada 10 segundos, com o registro da média desses valores a cada 5 minutos, em um total de 288 medidas ao dia. Esses dados são enviados para uma base (*Minimed Solutions CGMS Software® –Minimed, Inc.*) que faz a análise gráfica e estatística dos valores obtidos produzindo relatório das informações (18, 19). Esse tipo de monitorização pode auxiliar em diversas situações que possam provocar oscilações nos níveis glicêmicos, tais como ajuste terapêutico em pacientes com diabetes, em gestantes diabéticas e para o diagnóstico de hipoglicemias asintomáticas (especialmente noturnas) (20).

Quando comparada com as medidas convencionais de glicemia, o CGMS provê maior conhecimento da direção, magnitude, duração e freqüência das flutuações da glicemia (21). A diferença média absoluta entre o que é avaliado pelo sensor e pelas medidas usuais de glicemia é de 1.3 a 2.6 mmol/L, refletindo o atraso biológico que ocorre entre as concentrações de glicose no interstício e plasma. A sensibilidade e especificidade do CGMS para detector episódios hipoglicêmicos (<55 mg/dl), mostrou que o CGMS identifica corretamente 33% destes eventos e 96% fora da faixa de hipoglicemia.(22). O fato da acurácia ser muito melhor em faixas de normo e hiperglicemia favorece sua adequação para o presente projeto, em que a chance de hipoglicemia é mínima, tendo em vista os pacientes não fazerem uso de medicações anti-hiperglicêmicas. Apesar do CGMS ter sido usado para detectar hipo e hiperglicemia em pacientes com DM tipo 1 submetidos a atividade física intensa (23), poucos estudos avaliaram sua reprodutibilidade nesta situação.

Considerando-se a reprodutibilidade das glicemias avaliadas por glicemia capilar ou venosa em relação àquelas avaliadas pelo CGMS, poucos estudos abordam o problema durante atividade física. Em pacientes obesos com DM tipo 2, tanto a glicemia capilar venosa, como a avaliada pelo CGMS, foram reduzidas durante o exercício físico, conforme esperado, o que não ocorreu no grupo controle com as mesmas avaliações. O CGMS superestimou a glicemia nos grupos controle e diabético durante o exercício. O número de pontos fora do intervalo de confiança de 95% foi, no entanto, <5% em ambos os grupos, mostrando que a concordância entre os métodos durante o exercício é aceitável do ponto de vista estatístico. (24). Outro grupo que avaliou a reprodutibilidade do método, o fez em não diabéticos, comparando os sensores aplicados em tecido subcutâneo do abdômen e de antebraço à glicemia capilar e venosa. Os autores descrevem que a redução inicial da glicemia durante o exercício não foi completamente detectada com o uso de CGMS, tanto em sensores no antebraço como no abdômen. Durante a noite, os valores de glicemia avaliados pelo CGMS colocado em tecido subcutâneo do abdômen foram aproximadamente 20% menores do que aqueles avaliados pela glicemia capilar e medidos por sensores no antebraço (25). Portanto, a literatura ainda não respondeu de forma precisa o quanto a avaliação da glicemia por CGMS é reprodutível em pacientes com DM submetidos ao exercício físico.

Poucos estudos utilizaram o CGMS para avaliar o perfil glicêmico após atividade física em pacientes com diabetes. Cauza et al (26) avaliaram 15 pacientes com DMT2 submetidos a 4 meses de treinamento físico resistido ou aeróbico quanto ao perfil glicêmico (CGMS) 48h antes e após o treinamento, mostrando redução da glicemia média (15,6%) obtida com o CGMS apenas para os pacientes que foram submetidos ao treinamento aeróbico. Importante ressaltar que, apesar de não significativa, havia diferença na glicemia e índice de massa corporal basais dos 2 grupos estudados (maiores no grupo que foi submetido ao treinamento aeróbico) e não foi estudado um grupo controle. O seguimento de 10 desses pacientes por mais 4 meses com manutenção da atividade física aeróbica evidenciou melhoria adicional no perfil glicêmico (27).

A avaliação de efeitos agudos e subagudos do exercício físico aeróbico (cicloergômetro, 90% capacidade aeróbica máxima, 1 hora duração) foi investigada em pequeno grupo de pacientes com DMT2 através do CGMS mostrando redução da glicemia após o exercício e também de sua média em todo o dia após o exercício, o que não ocorreu no grupo controle. Outros autores realizaram estudo semelhante em DMT2 tratados com insulina. As excursões hiperglicêmicas foram diminuídas em 39% durante um período de 24h após uma sessão aguda de exercícios, mas a média da glicemia de 24h antes e após a sessão não foi diferente (28). Em pacientes com DMT1 o uso de CGMS mostrou uma freqüência elevada de hipoglicemias noturnas após exercício físico intenso aeróbico associado ao resistido (29).

Como bem conhecido e previamente descrito por nosso grupo em estudo com pacientes com DMT1, mesmo com diferentes tipos de exercício físico aplicados, para que se possam comparar suas respostas fisiológicas, a intensidade do exercício deve ser padronizada, o que pode ser feito através dos limiares de lactato individuais (30).

Tendo em vista o pequeno número de estudos citados na área, principalmente em relação ao uso do CGMS como forma de avaliar o perfil glicêmico durante e após uma sessão isolada de exercício físico, assim como sua reprodutibilidade em relação à glicemia capilar nesta situação específica, este estudo visa avaliar os efeitos agudos e subagudos de uma sessão de exercício físico aeróbico comparando aos efeitos de uma sessão de exercício físico combinado (aeróbico e resistido) em pacientes com DMT2 sem uso de medicações anti-hiperglicêmicas utilizando o CGMS como principal ferramenta na avaliação do perfil glicêmico.

**2 Objetivos**

**2.1 Geral**

Comparar os efeitos agudos e subagudos de uma sessão de exercício físico aeróbico *vs* de uma sessão de exercício físico combinado (aeróbico e resistido) em relação ao perfil glicêmico avaliado através do Sistema de Monitorização Contínua de Glicose (CGMS) em pacientes com DMT2.

**2.2 Específicos**

1. Avaliar o consumo máximo de oxigênio (VO_2_máx) através de ergoespirometria e a carga máxima para uma repetição através do teste de uma repetição máxima (1-RM) em pacientes com DMT2.
2. Analisar os resultados obtidos através do CGMS antes, durante e após uma sessão de exercício físico aeróbico e de uma sessão de exercício físico combinado (aeróbico e resistido), comparando com as medidas de glicemia capilar realizadas durante os dias em que o paciente se encontra monitorado pelo CGMS, para avaliar a concordância entre os métodos.

**3 Hipóteses**

Espera-se encontrar um maior benefício em relação ao controle glicêmico após uma sessão de exercício combinado quando comparado aos benefícios obtidos a partir de uma sessão de exercício aeróbico.

Em relação à reprodutibilidade do CGMS antes, durante e após cada sessão de exercício físico (aeróbico e combinado), comparado aos resultados das medidas de glicemia capilar realizadas durante os dias em que o paciente se encontra monitorado pelo CGMS, esperamos que haja concordância entre os métodos. A concordância entre os dois métodos citados já foi averiguada em estudo prévio (24), em situação semelhante.

**4 Materiais e Métodos**

**4.1 Delineamento da Pesquisa**

O delineamento do estudo será ensaio clínico randomizado, onde os pacientes serão distribuídos aleatoriamente em um dos seguintes grupos: exercício aeróbico na 1ª semana e exercício combinado na 2ª semana (Grupo A), ou exercício combinado na 1ª semana e exercício aeróbico na 2ª semana (Grupo B). Os pacientes serão sorteados somente quanto à ordem de realização de cada tipo de exercício físico.

**4.2 Pacientes**

Serão selecionados pacientes com DMT2 (diagnóstico após os 35 anos), sexo masculino, com idade inferior a 65 anos, com pelo menos 3 anos de duração da patologia, em tratamento com dieta apenas, sem uso de drogas anti-hiperglicêmicas ou insulina, com descontrole metabólico leve a moderado (glicemia de jejum entre 125 e 200 mg/dl, HbA1c entre 7,0 e 8,5%).

A amostra consistirá de 18 pacientes. Esse tamanho de amostra é suficiente para detectar uma diferença de aproximadamente 3,0 quanto ao número de episódios hiperglicêmicos, considerando um poder estatístico de 80% (erro β = 0,2) e um erro α = 0,05. O cálculo do tamanho da amostra foi realizado com base nos dados de Praet et al (24), que demonstraram diminuição do número médio de episódios hiperglicêmicos de 7.6 ± 1.4 para 4.6 ± 1.1 comparando 24 horas depois de uma sessão aguda de exercício resistido vs valores de 24h do mesmo dia do exercício.

Consideramos que o número de pacientes necessário poderá ser obtido com certa facilidade de acordo com medidas adequadas para sua busca, tais como colocação de anúncio em jornal de grande circulação ou contato com colegas que trabalham em postos de saúde de atenção primária e secundária onde o tipo de paciente que necessitamos é mais freqüentemente atendido. Estas estratégias serão desenvolvidas durante projeto piloto e a mais efetiva será empregada.

**4.3 Protocolo**

Os candidatos ao estudo seguirão um protocolo composto por 4 etapas. 1ª etapa: serão convidados, assinarão termo de consentimento informado, responderão um questionário específico e serão submetidos a exame físico sumário, coleta de sangue em jejum e de amostra de urina, eletrocardiograma de repouso e exame de fundo de olho. 2ª etapa: realizarão ergoespirometria e teste de 1-RM para a adequada avaliação de sua capacidade funcional e prescrição da sessão de exercício. 3ª etapa: distribuição aleatória dos pacientes em um dos seguintes grupos: exercício aeróbico na 1ª semana e exercício combinado na 2ª semana (Grupo A), ou exercício combinado na 1ª semana e exercício aeróbico na 2ª semana (Grupo B).

**4.3.1 Seleção: 1ª etapa**

Os pacientes serão selecionados a partir de interesse após divulgação através de jornal. O primeiro contato com o paciente será por telefone, quando será feita uma rápida entrevista na tentativa de excluir aqueles com critérios de exclusão (Anexo I). Os pacientes pré-selecionados serão informados sobre o estudo e convidados a participar. A primeira visita do paciente ao HCPA será agendada e nesse momento as questões referentes aos critérios de exclusão, abordadas por telefone, serão novamente avaliadas. Com a respectiva assinatura do termo de consentimento informado (Anexo II), os pacientes responderão a um questionário e serão submetidos a um exame físico sumário (Anexo III). Serão coletadas amostras de sangue e urina em jejum para glicemia, HbA1c, insulinemia, creatinina, colesterol e frações, triglicerídeos, e índice proteína/creatinina. Um eletrocardiograma de repouso e um exame de fundo de olho sob midríase também serão realizados nessa etapa.

São critérios de exclusão: diabetes descompensado (glicemia jejum>200 mg/dl, HbA1c>8,5%), retinopatia diabética proliferativa, neuropatia autonômica grave, neuropatia periférica grave, amputações, vasculopatia, hipertensão arterial não controlada, cardiopatia isquêmica sintomática, insuficiência cardíaca, nefropatia diabética clínica (índice proteína-creatinina>0,2) e insuficiência renal cônica (creatinina>1,4 mg/dl).

Os pacientes que se encaixarem nos critérios de inclusão e não tiverem critérios de exclusão serão encaminhados para a segunda etapa do projeto.

**4.3.2 Testes de Exercícios Aeróbico e Resistido: 2ª etapa**

**Ergoespirometria.** Será realizada conforme previamente descrito (31).

A capacidade funcional máxima será mensurada através de um teste cardiopulmonar de exercício máximo com análise de gases expirados, em esteira Inbramed® KT 10200 (Porto Alegre, Brasil), a qual permite adquirir dados de indivíduos com até 150 Kg de peso corporal, com velocidade variável entre 0 a 16 Km/h (0 a 10 Mph) e elevação de rampa entre 0 e 26%.

O protocolo iniciará a uma velocidade de 2.4 Km/h e inclinação de 2%. O aumento na velocidade ocorrerá a cada 20 segundos (0.1 a 0.2 Km/h) e o da inclinação a cada 60 segundos (0.1 a 0.2 %), com objetivo de alcançar a fadiga em um tempo médio de 10 minutos. No período pré-teste propriamente dito, o indivíduo caminhará entre 2 e 4 minutos para se adaptar a esteira. O avaliado terá seu monitoramento cardíaco obtido através do traçado eletrocardiográfico a cada 1 minuto (Nikon Kohden Corporation®, Tókio, Japão). A mensuração da PA será realizada com esfigmomanômetro a cada minuto de teste. O consumo de oxigênio (VO_2_), produção de dióxido de carbono (VCO_2_), ventilação minuto (V_E_), relação de troca gasosa (R), equivalentes ventilatórios de oxigênio (V_E_/VO_2_) e de dióxido de carbono (V_E_/VCO_2_) serão mensurados, por 20 segundos, através da análise efetuada pelo Total Metabolic Analysis Sistem – TEEM 100® (AeroSport®, Ann Arbor, USA), previamente validado (32).

O VO_2_ de pico será considerado o máximo consumo alcançado nos últimos segundos do exercício. As medidas deverão ser obtidas com o avaliado na posição ereta em repouso por cinco minutos, durante todo o período progressivo do exercício e sentado, ao longo dos primeiros 3 minutos de recuperação.

O teste será interrompido por resposta hipertensiva em baixa carga, atingir níveis críticos de PA (acima de 240/ 140 mmHg), presença de arritmia grave, presença de alterações eletrocardiográficas sugestivas de isquemia miocárdica, acompanhada ou não de dor anginosa típica, dor em membros inferiores sugestivos de insuficiência vascular arterial, queda infra-esforço da PA, pedido do indivíduo, exaustão e qualquer situação que o ergoespirometrista julgue relevante.

Antes e após a ergoespirometria, os pacientes realizarão uma medida de glicemia capilar (Advantage, Roche) e terão seus sinais vitais verificados.

**Teste de 1 repetição máxima (Teste de 1-RM).** Para avaliação da força máxima nos exercícios resistidos iremos aplicar – ao início do estudo – o teste de 1-RM para cada um dos movimentos que serão posteriormente realizados na sessão de exercício resistido. Esse teste consiste na realização tecnicamente adequada de exercícios resistidos com a máxima quantidade de carga. Os procedimentos do teste incluem aquecimentos livres (movimentação livre dos membros que serão exercitados) e específicos (aquecimento executado diretamente com o movimento que será testado, porém com cargas leves a moderadas). Os movimentos testados serão idênticos aos que posteriormente comporão a sessão submáxima de exercício resistido, sendo eles: pressão de pernas, extensão de joelhos, rosca bíceps (flexão de cotovelos), tríceps testa (extensão de cotovelos), crucifixo frontal (flexão horizontal de ombros) e crucifixo invertido (extensão horizontal de ombros). Os testes serão realizados após liberação médica para tal procedimento, e 2 profissionais de educação física com experiência em treinamento resistido irão conduzir as avaliações. É válido ressaltar que o teste de 1-RM é largamente aplicado em estudos com treinamento de força e tem sido utilizado em diferentes populações (33-35).

Antes e após o teste de 1RM, os pacientes realizarão uma medida de glicemia capilar (Advantage, Roche) e terão seus sinais vitais verificados.

**4.3.3 Distribuição Aleatória: 3ª etapa**

Logo após concluídas a 1ª e a 2ª etapas, os pacientes serão distribuídos aleatoriamente (somente quanto à ordem de realização de cada tipo de exercício físico) em Grupo A – exercício aeróbico na 1ª semana e exercício combinado na 2ª semana dessa etapa e Grupo B – exercício combinado na 1ª semana e exercício aeróbico na 2ª semana dessa etapa. Ou seja, 9 dos 18 pacientes realizarão o exercício aeróbico na 1ª semana e o exercício combinado na 2ª semana e os outros 9 pacientes realizarão os exercícios na ordem inversa, exercício combinado na 1ª semana e exercício aeróbico na 2ª semana.

**4.3.4 Sessões de Exercício: 4ª etapa**

Os pacientes realizarão sessões submáximas de exercício aeróbico e exercício combinado, durante as quais serão monitorados através do CGMS. Essa etapa se inicia na semana seguinte à 3ª etapa. Cada condição de exercício (aeróbico ou combinado) será realizada em uma semana diferente, sendo que alguns pacientes realizarão primeiro a sessão submáxima de exercício físico aeróbico e na semana seguinte a sessão submáxima de exercício físico combinado e outros pacientes realizarão essas sessões de exercícios na ordem inversa, de forma aleatória. Portanto, serão necessárias duas semanas para que cada paciente conclua a 4ª etapa. O CGMS será instalado 1 dia antes do dia da sessão de exercício e retirado 1 dia após, tendo em vista que o tempo de monitorização que o equipamento permite realizar é de 36h.

**Sessão Submáxima de Exercício Físico Aeróbico.** A sessão submáxima de exercício aeróbico será realizada no Laboratório de Fisiopatologia do Exercício (LaFiEx). A sessão terá a duração total 50 minutos, com 5 minutos iniciais para familiarização e ajuste da carga no cicloergômetro, 40 minutos de exercício aeróbico à intensidade de 70% do consumo máximo de oxigênio, e 5 minutos finais de alongamento e relaxamento. A intensidade do exercício aeróbico será quantificada através da FC correspondente ao valor de 70% do consumo máximo de oxigênio obtido através da Ergospirometria, realizada pelo paciente na 2ª etapa do protocolo. A freqüência cardíaca, pressão arterial e glicemia dos indivíduos serão monitoradas continuamente durante a prática do exercício físico.

Antes e após a sessão de exercício físico aeróbico, os pacientes realizarão uma medida de glicemia capilar (Advantage, Roche) e terão seus sinais vitais verificados.

Sumário:

1º dia: O CGMS é colocado pela manhã.

2º dia: Sessão submáxima de exercício aeróbico (40 minutos de exercício aeróbico no cicloergômetro à intensidade de 70% do VO_2_máx).

3º dia: Mantém-se o CGMS para avaliar os efeitos pós-exercício.

4º dia: O CGMS é retirado (mantido pelo menos por 72h).

**Sessão Submáxima de Exercício Físico Combinado (aeróbico e resistido).** A sessão submáxima de exercício resistido será realizada no Laboratório de Fisiopatologia do Exercício (LaFiEx). A sessão terá a duração total 50 minutos, onde serão realizados 20 minutos de exercício aeróbico e 20 minutos de exercício resistido. Nos 5 minutos iniciais serão realizados exercícios de aquecimento, seguidos de 20 minutos de exercício aeróbico à intensidade de 70% do consumo máximo de oxigênio (o qual será previamente avaliado na Ergoespirometria). A intensidade do exercício aeróbico será quantificada através da FC correspondente ao valor de 70% do consumo máximo de oxigênio obtido através da Ergospirometria. Após o exercício aeróbico, serão realizados 20 minutos de exercícios resistidos, seguidos de 5 minutos finais de alongamento e relaxamento.

Portanto, nessa sessão, além do exercício aeróbico em cicloergômetro também serão realizados exercícios resistidos que envolvem os membros superiores e os membros inferiores. A intensidade utilizada será de 65% de 1-RM (sendo esta última previamente avaliada através do teste de 1 repetição máxima, realizado pelo paciente na 2ª etapa do protocolo) e 3 séries serão executadas para cada movimento, sendo que cada série compreenderá 12 repetições. O tempo padronizado para cada repetição será de 4 segundos (2 segundos na fase concêntrica e 2 segundos na fase excêntrica do exercício), e os intervalos entre cada série serão de 45 a 60 segundos. A freqüência cardíaca, pressão arterial e glicemia dos indivíduos serão monitoradas continuamente durante a prática do exercício físico. A intensidade do exercício resistido será de 65% da carga máxima (em Kg) encontrada através do teste de uma repetição máxima realizado para todos os exercícios resistidos a serem trabalhados.

Antes e após a sessão de exercício físico combinado (aeróbico e resistido), os pacientes realizarão uma medida de glicemia capilar (Advantage, Roche) e terão seus sinais vitais verificados.

Sumário:

1º dia: O CGMS é colocado pela manhã.

2º dia: Sessão submáxima de exercício combinado (20 minutos de exercício aeróbico no cicloergômetro à intensidade de 70% do VO_2_máx seguidos de 20 minutos de exercícios resistidos à intensidade de 65% de 1-RM).

3º dia: Mantém-se o CGMS para avaliar os efeitos pós-exercício.

4º dia: O CGMS é retirado (mantido pelo menos por 72h).

O CGMS (Sistema Monitorização Continua de Glicose) consiste na introdução de sensores no tecido subcutâneo, através dos quais é possível registrar as glicemias ao longo do dia de maneira ininterrupta (17-19). As sessões submáximas de exercício físico aeróbico e de exercício físico combinado serão realizadas em semanas distintas. Os sensores serão introduzidos no tecido subcutâneo dos pacientes um dia antes de cada sessão de exercício e serão retirados no dia seguinte à sessão.

O CGMS terá a função de avaliar os efeitos agudos e subagudos de uma sessão de exercício físico aeróbico e de uma sessão de exercício físico combinado (aeróbico e resistido) em relação ao perfil glicêmico. Serão considerados efeitos agudos as respostas fisiológicas ocorridas enquanto o exercício é executado e efeitos subagudos os efeitos residuais de manifestações fisiológicas ocorridas durante o exercício que decrescem progressivamente depois que o mesmo é interrompido (36).

**4.4 Perfil glicêmico**

Será avaliado através do CGMS (3 dias), da glicemia e da HbA1c.

**4.5 Medidas bioquímicas**

Sangue venoso para medidas bioquímicas (colesterol e frações, glicemia, creatinina, hemograma), será coletado em jejum de 12 horas no dia que o paciente comparecer para as avaliações. O colesterol plasmático e os triglicerídeos serão analisados usando k*its* enzimáticos. O HDL-c será isolado usando o método heparina-2M Mncl2 e medidos com o mesmo kit enzimático usado para o colesterol plasmático total. O LDL-c será estimado pela fórmula de Friedewald. Para análise da glicemia, serão usados *kits* disponíveis comercialmente. O hemograma será analisado em contador automático, modelo 818 da *AVL*. A insulinemia será medida usando *kits* comerciais.

O HOMA-IR (Homeostasis model assessment) será calculado como o produto da insulinemia de jejum (um/ml) e glicose plasmática de jejum (mmol/l) dividido por 22.5. O HOMA-IR provê uma forma útil de avaliar a resistência à insulina em estudos epidemiológicos (37).

O IPC, o qual corresponde à razão entre proteinúria e creatininúria em urina de amostra, e a HbA1c também serão analisados.

**4.6 Considerações éticas**

O projeto será submetido ao Comitê de Ética em Pesquisa do HCPA.

**4.7 Análise estatística**

Será realizada através do recurso do programa *Statistical Package for Social Sciences* (SPSS). Para todos os resultados serão observadas as características da amostra por média e desvio padrão. Na sua comparação será utilizada análise de variância para medidas repetidas (ANOVA) e *post hoc* com comparação Bonferroni entre os grupos 1 e 2.

**5 Orçamento**

|  | **MATERIAL DE CONSUMO** |  |  |
| --- | --- | --- | --- |
| **Quantidade** | **Descrição** | **Valor unitário** | **Valor total** |
| 1 | Folhas A4 (500) | 10,00 | 10,00 |
| 90 | Fotocópias Anexo I, TCLE e III | 0,15 | 13,50 |
| 36 | CGMS (sensores)* | 146,00 | 5.256,00 |
| 18 | Ergoespirometria | 30,00 | 540,00 |
| 18 | Glicemia | 1,85 | 33,30 |
| 18 | HbA1c – Hemoglobina Glicada | 7,86 | 141,48 |
| 18 | Creatinina no sangue | 1,85 | 33,30 |
| 18 | Proteína (amostra urina) – por eletroforese | 4,44 | 79,92 |
| 18 | Creatinina (amostra urina) | 1,85 | 33,30 |
| 18 | Colesterol total | 1,85 | 33,30 |
| 18 | Colesterol HDL | 3,51 | 63,18 |
| 18 | Triglicerídeos | 3,51 | 63,18 |
| 18 | Insulinemia | 8,84 | 159,12 |
| 252 | Passagens | 3,00 | 756,00 |
| 576 | Fitas reagentes (glicemia capilar)** | 1,00 | 576,00 |
| **TOTAL** |  |  | **7.791,58** |

* Sensores de CGMS adquiridos por projeto FAPERGS e disponíveis no Serviço de Endocrinologia do HCPA **5.256,00**

** Fitas reagentes serão doadas pela Advantage, Roche **576,00**

**Financiamento FIPE**  **1.959,00**

**6** Cronograma

|  | **1^o^ sem 2008** | **2^o^ semestre 2008** | | **1º semestre 2009** | | **2^o^ semestre 2009** | | **1^o^ sem 2010** |
| --- | --- | --- | --- | --- | --- | --- | --- | --- |
|  | **2^o^ tri** | **1^o^ tri** | **2^o^ tri** | **1^o^ tri** | **2^o^ tri** | **1^o^ tri** | **2^o^ tri** | **2^o^ tri** |
| **Rev bibliográfica** | X | X | X | X | X | X | X | X |
| **Redação projeto** | X |  |  |  |  |  |  |  |
| **Comitê ética** |  | X |  |  |  |  |  |  |
| **Treinamento equipe** |  | X | X | X | X |  |  |  |
| **Coleta de dados** |  | X | X | X | X | X |  |  |
| **Banco de dados** |  |  |  | X | X | X | X |  |
| **Análise estatística** |  |  |  |  | X | X | X |  |
| **Redação tese** |  |  |  |  |  | X | X | X |
| **Defesa tese** |  |  |  |  |  |  |  | X |
| **Publicação** |  |  |  |  |  |  |  | X |

**Referências Bibliográficas**

1. **Brasil MdS, Secretaria de Políticas de Saúde, Departamento de Ações Programáticas Estratégicas.** 2001 Plano de reorganização da atenção à hipertensão arterial e ao diabete melito: hipertensão arterial e diabete melito / Departamento de Ações Programáticas Estratégicas. Brasília: Ministério da Saúde:104p

2. **Schaan BD, Harzheim E, Gus I** 2004 [Cardiac risk profile in diabetes mellitus and impaired fasting glucose]. Rev Saude Publica 38:529-36

3. **Knowler WC, Barrett-Connor E, Fowler SE, et al.** 2002 Reduction in the incidence of type 2 diabetes with lifestyle intervention or metformin. N Engl J Med 346:393-403

4. **Boule NG, Kenny GP, Haddad E, Wells GA, Sigal RJ** 2003 Meta-analysis of the effect of structured exercise training on cardiorespiratory fitness in Type 2 diabetes mellitus. Diabetologia 46:1071-81

5. 2008 Standards of medical care in diabetes--2008. Diabetes Care 31 Suppl 1:S12-54

6. **Haskell WL, Lee IM, Pate RR, et al.** 2007 Physical Activity and Public Health: Updated Recommendation for Adults from the American College of Sports Medicine and the American Heart Association. Med Sci Sports Exerc 39:1423-1434

7. **Boule NG, Haddad E, Kenny GP, Wells GA, Sigal RJ** 2001 Effects of exercise on glycemic control and body mass in type 2 diabetes mellitus: a meta-analysis of controlled clinical trials. Jama 286:1218-27

8. **Wallberg-Henriksson H, Rincon J, Zierath JR** 1998 Exercise in the management of non-insulin-dependent diabetes mellitus. Sports Med 25:25-35

9. **Zachwieja JJ, Toffolo G, Cobelli C, Bier DM, Yarasheski KE** 1996 Resistance exercise and growth hormone administration in older men: effects on insulin sensitivity and secretion during a stable-label intravenous glucose tolerance test. Metabolism 45:254-60

10. **Zinman B, Ruderman N, Campaigne BN, Devlin JT, Schneider SH** 2004 Physical activity/exercise and diabetes. Diabetes Care 27 Suppl 1:S58-62

11. **Williams MA, Haskell WL, Ades PA, et al.** 2007 Resistance exercise in individuals with and without cardiovascular disease: 2007 update: a scientific statement from the American Heart Association Council on Clinical Cardiology and Council on Nutrition, Physical Activity, and Metabolism. Circulation 116:572-84

12. **Nelson ME, Rejeski WJ, Blair SN, et al.** 2007 Physical Activity and Public Health in Older Adults: Recommendation from the American College of Sports Medicine and the American Heart Association. Med Sci Sports Exerc 39:1435-1445

13. **Castaneda C, Layne JE, Munoz-Orians L, et al.** 2002 A randomized controlled trial of resistance exercise training to improve glycemic control in older adults with type 2 diabetes. Diabetes Care 25:2335-41

14. **Dunstan DW, Daly RM, Owen N, et al.** 2002 High-intensity resistance training improves glycemic control in older patients with type 2 diabetes. Diabetes Care 25:1729-36

15. **Sigal RJ, Kenny GP, Boule NG, et al.** 2007 Effects of aerobic training, resistance training, or both on glycemic control in type 2 diabetes: a randomized trial. Ann Intern Med 147:357-69

16. 2007 Standards of medical care in diabetes--2007. Diabetes Care 30 Suppl 1:S4-S41

17. **Boland E, Monsod T, Delucia M, Brandt CA, Fernando S, Tamborlane WV** 2001 Limitations of conventional methods of self-monitoring of blood glucose: lessons learned from 3 days of continuous glucose sensing in pediatric patients with type 1 diabetes. Diabetes Care 24:1858-62

18. **Oliveira CH, Berger K, Souza SC, et al.** 2005 [Continuous glucose monitoring: a critical appraisal after one year experience]. Arq Bras Endocrinol Metabol 49:983-90

19. **Maia FF, Araujo LR** 2005 [Accuracy, effect on insulin therapy and glycemic control and complications of the continuous glucose monitoring system in type 1 diabetic patients]. Arq Bras Endocrinol Metabol 49:563-8

20. **Hay LC, Wilmshurst EG, Fulcher G** 2003 Unrecognized hypo- and hyperglycemia in well-controlled patients with type 2 diabetes mellitus: the results of continuous glucose monitoring. Diabetes Technol Ther 5:19-26

21. **Rebrin K, Stell G M, Antwerp W P V, Mastototaro J J** 1999 Subcutaneous glucose predicts plasma glucose independent of insulin: implications for continuous monitoring. Am. J. Physiol. 40:561-71

22. **Guerci B, Floriot M, Bohme P, et al.** 2003 Clinical performance of CGMS in type 1 diabetic patients treated by continuous subcutaneous insulin infusion using insulin analogs. Diabetes Care 26:582-89

23. **Carr J M, Corcoran M H** 2002 Continuous glucose monitoring in marathon runners with type 1 diabetes during the 2001 Chicago marathon. Diabetes 51:988

24. **MacDonald AL, Philp A, Harrison M, Bone AJ, Watt PW** 2006 Monitoring exercise-induced changes in glycemic control in type 2 diabetes. Med Sci Sports Exerc 38:201-7

25. Dobeln A V, Adamson U, Lins P E 2005 Nocturnal differences in subcutaneous tissue glucose between forearm and abdominal sites during continuous glucose monitoring in normal subjects Diabetes Metab. 31:347-52

26. **Cauza E, Hanusch-Enserer U, Strasser B, Kostner K, Dunky A, Haber P** 2005 Strength and endurance training lead to different post exercise glucose profiles in diabetic participants using a continuous subcutaneous glucose monitoring system. Eur J Clin Invest 35:745-51

27. **Cauza E, Hanusch-Enserer U, Strasser B, Kostner K, Dunky A, Haber P** 2006 The metabolic effects of long term exercise in Type 2 Diabetes patients. Wien Med Wochenschr 156:515-9

28. **Praet SF, Manders RJ, Lieverse AG, et al.** 2006 Influence of acute exercise on hyperglycemia in insulin-treated type 2 diabetes. Med Sci Sports Exerc 38:2037-44

29. **Iscoe KE, Campbell JE, Jamnik V, Perkins BA, Riddell MC** 2006 Efficacy of continuous real-time blood glucose monitoring during and after prolonged high-intensity cycling exercise: spinning with a continuous glucose monitoring system. Diabetes Technol Ther 8:627-35

30. **Bertoluci MC, Friedman G, Schaan BD, Ribeiro JP, Schmid H** 1993 Intensity-related exercise albuminuria in insulin dependent diabetic patients. Diabetes Res Clin Pract 19:217-25

31. **Camargo MD, Stein R, Ribeiro JP, Schvartzman PR, Rizzatti MO, Schaan BD** 2007 Circuit weight training and cardiac morphology: A trial with Magnetic Resonance Imaging. Br J Sports Med

32. **Novitsky S, Segal KR, Chatr-Aryamontri B, Guvakov D, Katch VL** 1995 Validity of a new portable indirect calorimeter: the AeroSport TEEM 100. Eur J Appl Physiol Occup Physiol 70:462-7

33. **Shaw CE, McCully KK, Posner JD** 1995 Injuries during the one repetition maximum assessment in the elderly. J Cardiopulm Rehabil.;15:283-7.

34. **Casaburi R, Bhasin S, Cosentino L, Porszasz J, Somfay A, Lewis MI, Fournier M, Storer TW** 2004 Effects of testosterone and resistance training in men with chronic obstructive pulmonary disease. Am J Respir Crit Care Med 170:870-8.

35. **Rooks DS, Silverman CB, Kantrowitz FG** 2002 The effects of progressive strength training and aerobic exercise on muscle strength and cardiovascular fitness in women with fibromyalgia: a pilot study. Arthritis Rheum 47:22-8.

36. **Nobrega, ACL** 2005 The subacute Effects of Exercise: Concep, Characteristics, and Clinical Implications. Exerc Sport Sci 33:84-7

37. **Matthews DR, Hosker JP, Rudenski AS, Naylor BA, Treacher DF, Turner RC** 1985 Homeostasis model assessment: insulin resistance and beta-cell function from fasting plasma glucose and insulin concentrations in man. Diabetologia 28:412-9

**ANEXO I: CRITÉRIOS DE EXCLUSÃO - ENTREVISTA POR TELEFONE**

Nome paciente: _____________________________________________________________________

N^o^ registro HCPA: ___________________

Data: _________ / _________ / _________

Se o paciente responder **“Sim”** para qualquer uma das perguntas abaixo, está automaticamente excluído da pesquisa. Quanto às questões que o paciente não souber informar, serão avaliadas na etapa a seguir (ex: não sabe HbA1c recente, não fez fundo de olho recente, etc).

**1-** Diagnóstico antes dos 35 anos (1) Sim (2) Não

**2-** Duração da patologia < 3 anos (1) Sim (2) Não

**3-** Idade > 65 anos ou < 35 anos (1) Sim (2) Não

**6-** Uso de insulina (1) Sim (2) Não

**7-** Glicemia de jejum > 200 ou < 125 (1) Sim (2) Não

**8-** HbA1c > 8,5% (1) Sim (2) Não

**9-** Retinopatia diabética proliferativa (1) Sim (2) Não

**10-** Neuropatia autonômica grave (1) Sim (2) Não

**11-** Neuropatia periférica grave (1) Sim (2) Não

**12**- Vasculopatia (1) Sim (2) Não

**13**- Amputação (1) Sim (2) Não

**14-** Nefropatia diabética clínica

(índice proteína-creatinina > 0,2) (1) Sim (2) Não

**15-** Insuficiência renal cônica

(creatinina > 1,4 mg/dl) (1) Sim (2) Não

**16-** Insuficiência cardíaca (1) Sim (2) Não

**17-** Cardiopatia isquêmica sintomática (1) Sim (2) Não

**18-** Hipertensão arterial não controlada (1) Sim (2) Não

**19-** Dor torácica (1) Sim (2) Não

**20-** Alteração no ECG de repouso (1) Sim (2) Não

**21-** História de AVE (1) Sim (2) Não

**22-** Infarto Agudo do Miocárdio (1) Sim (2) Não

**23-** CRM (1) Sim (2) Não

**24-** ACTP (1) Sim (2) Não

( ) Incluído ( ) Excluído

# ANEXO II: TERMO DE CONSENTIMENTO LIVRE E ESCLARECIDO

# Projeto: Efeitos Agudos e Subagudos do Exercício Físico Aeróbico e Aeróbico/Resistido em Pacientes com Diabetes Tipo 2 avaliados através do

# Sistema de Monitorização Contínua de Glicose

Neste documento iremos explicar os objetivos e cada etapa do estudo para qual o senhor (a) está sendo convidado.

A prática de exercícios físicos regulares traz uma série de benefícios para pacientes com Diabetes Mellitus Tipo 2 (DMT2), tais como redução do risco de doenças cardíacas, osteoporose, obesidade, etc. A atividade física regular melhora a sensibilidade à insulina prejudicada pela doença, auxiliando em melhorar o controle da glicose. O efeito de maior sensibilidade à insulina induzido por uma única sessão de exercício físico aeróbico pode durar entre 24 e 72h, dependendo da sua intensidade e duração.

Sendo assim, através desta pesquisa, buscamos avaliar os efeitos agudos e subagudos de uma sessão de exercício físico aeróbico comparando aos efeitos de uma sessão de exercício físico combinado (aeróbico + musculação) em pacientes com DMT2 sem uso de medicações para o diabetes, utilizando o Sistema de Monitorização Continua de Glicose como principal ferramenta na avaliação do perfil glicêmico.

Para isto, precisaremos que o senhor (a) participe de três etapas de nosso estudo:

**1ª etapa:** a) responder a um questionário, b) submeter-se a um exame físico, c) coletar amostras de sangue e de urina em jejum (será puncionada uma veia do antebraço para a coleta de 12 mL de sangue – 2 colheres de sopa – para que se possam realizar os exames laboratoriais), d) realizar um eletrocardiograma de repouso, e) realizar um exame de fundo de olho. Realização dessa etapa: um encontro na 1ª semana. Duração da etapa: 2 horas

**2ª etapa:** realizar: a) um teste máximo de exercício em bicicleta ergométrica (ergoespirometria), para a partir daí podermos prescrever de maneira adequada a sessão de exercício aeróbico a ser realizada, b) um teste máximo para avaliar sua força em determinados exercícios de musculação, para a partir daí podermos prescrever de maneira adequada a sessão de exercícios de musculação a ser realizada. Realização dessa etapa: um encontro na 2ª semana. Duração da etapa: 2 horas

**3ª etapa:** realizar: a) uma sessão de 40 minutos de exercício físico aeróbico em bicicleta ergométrica, b) uma sessão de 40 minutos de exercício físico combinado (20 minutos de exercício físico aeróbico em bicicleta ergométrica + 20 minutos de exercício físico de musculação em aparelhos e com pesos livres). Realização dessa etapa: três encontros na 3ª semana (1º encontro: colocação dos sensores, duração: 1 hora; 2º encontro: realização de uma das sessões de exercício, duração: 1 hora; 3º encontro: retirada dos sensores, duração: 1 hora) e três encontros na 4ª semana (1º encontro: colocação dos sensores, duração: 1 hora; 2º encontro: realização da outra sessão de exercício, duração: 1 hora; 3º encontro: retirada dos sensores, duração: 1 hora). Duração da etapa: 6 horas (1 hora por encontro, sendo que são três encontros por semana).

O Sistema de Monitorização Continua de Glicose consiste na introdução de sensores no tecido subcutâneo (camada localizada abaixo da derme), através dos quais é possível registrar as glicemias ao longo do dia. Este sensor é aplicado com um aplicador que contém uma agulha que é inserida na pele do paciente. Esta agulha é então retirada, ficando apenas um cateter plástico (tubo fino, macio e flexível) sob a pele. Os sensores serão introduzidos um dia antes de cada sessão de exercício e serão retirados no dia seguinte à sessão.

Os riscos ou desconfortos que o senhor (a) ficará exposto serão mínimos, incluindo dor e hematoma (mancha roxa na pele) no local de coleta de sangue e no local onde será introduzido o sensor para a monitorização da glicose. Estes exames são amplamente empregados em pesquisas e já foram realizadas várias vezes por nossa equipe, sendo extremamente seguros. Os testes de exercício bem como as sessões de exercício serão conduzidos por equipe treinada e todos ocorrerão em nosso laboratório, o qual é um ambiente preparado para reduzir riscos aos pacientes, visto que está localizado dentro do Hospital de Clínicas de Porto Alegre, possuindo equipamentos de monitorização dos pacientes (termômetro, aparelhos de pressão, eletrocardiograma) e cardiodesfibrilador automático (equipamento [eletrônico](http://pt.wikipedia.org/wiki/Eletrônica) cuja função é reverter paradas cardíacas) em caso de urgências.

Ao concluir todas as etapas de nosso estudo, o senhor (a) obterá informações detalhadas sobre a sua saúde e aspectos relacionados ao Diabetes, tais como: exame de sangue, eletrocardiograma de repouso, exame de fundo de olho, teste de esforço aeróbico (ergoespirometria), teste de força muscular e avaliação detalhada do controle da glicose.

Em nenhum momento seu nome ou qualquer informação sobre a sua saúde será fornecido para qualquer outra pessoa que não seja um dos pesquisadores. As informações serão utilizadas somente para fins de pesquisa, sendo garantida a confidencialidade dos dados. O (A) senhor (a) tem direito de recusar-se a participar deste estudo e sua decisão não influenciará em nada no seu atendimento no HCPA.

Pelo Termo de Consentimento Livre e Esclarecido declaro que autorizo minha participação neste projeto de pesquisa. Fui informado (a), de forma clara e detalhada, livre de qualquer forma de constrangimento e coerção, sobre os objetivos desse estudo. Fui igualmente informado (a):

- da garantia de receber resposta a qualquer pergunta ou esclarecimento a qualquer dúvida acerca dos procedimentos, riscos, benefícios e outros assuntos relacionados com a presente pesquisa;

- da liberdade de retirar meu consentimento, a qualquer momento, e deixar de participar do estudo, sem que isso me traga prejuízos;

- da garantia de que não serei identificado quando da divulgação dos resultados e que as informações obtidas serão utilizadas apenas para fins científicos vinculados ao presente projeto de pesquisa;

- do compromisso de proporcionar informação atualizada obtida durante o estudo, ainda que esta possa afetar a minha vontade em continuar participando;

- do consentimento e liberação do telefone residencial para eventual contato com a finalidade informativa sobre o meu estado clínico de saúde.

Eu ________________________________________ acredito ter sido suficientemente informado a respeito da pesquisa que tem por objetivo avaliar os efeitos agudos e subagudos de uma sessão de exercício físico aeróbico comparando aos efeitos de uma sessão de exercício físico combinado (aeróbico + musculação) em pacientes com DMT2, utilizando o Sistema de Monitorização Continua de Glicose. Eu discuti com a equipe pesquisadora sobre a minha decisão de participar desta pesquisa. Ficam claros para mim, quais são os propósitos do estudo, os procedimentos a serem realizados, seus desconfortos, riscos, as garantias de confidencialidade e de esclarecimento permanente. Ficou claro também que minha participação é isenta de despesas e de remuneração, e que a qualquer momento posso desistir do exame sem prejuízo ao acesso e tratamento neste hospital.

__________________________________________________

Assinatura do participante

Pesquisador executor: Franciele Ramos Figueira

Telefone: (51) 96811968

__________________________________________________

Assinatura do pesquisador executor

Responsável pelo projeto: Profa. Dra. Beatriz D’Agord Schaan

Telefone: (51) 21 01 81 27

**ANEXO III: QUESTIONÁRIO E EXAME FÍSICO SUMÁRIOS**

**Perfil sócio-demográfico** **Data: ______/______/______**

Nome do paciente: __________________________________________________________________

Endereço: _________________________________________________________________________

Fone: ________ ________ Cidade: ____________________ Estado: ____________________

Fone (contatos): ____________________________________________________________________

N^o^ registro HCPA: ______________________

Data de nascimento: ______ / ______ / ______

**1-** Idade: _____________ anos

**2-** Gênero (1) Masculino (2) Feminino

**3-** Raça (1) Negra (2) Branca (3) Indígena (4) Amarela

**4-** Escolaridade: _______ anos de estudo completados ( ) analfabeto

**5-** Tempo de duração do DMT2: **____________** anos

**6-** PA (1ª medida sentado): ________/________ (mmHg)

**7-** PA (2ª medida sentado): ________/________ (mmHg)

**8-** FC repouso (sentado): _________ bpm

**9-** PA (1ª medida em pé): ________/________ (mmHg)

**10-** FC repouso (em pé): ________ bpm

**11-** Peso: ________ Kg

**12-** Altura: ________ cm

**13-** Índice massa corporal: (IMC) ___________

**14-** Circunferência abdominal: _____________

**15-** Circunferência quadril: ________________

**16-** Glicemia capilar na chegada: ___________ (mg/dL) ( ______ horas de jejum)

**17-** Hb1Ac: _____________________

**18-** Creatinina: __________________

**19-** Colesterol total: ______________

**20-** Colesterol HDL: ______________

**21-** Insulinemia: _________________

**22-** Triglicerídeos: _______________

**23-** Proteína (amostra urina): _____________________

**24-** Creatinina (amostra urina): ___________________

**25-** Exame de fundo de olho realizado por oftalmologista HCPA: _____________________________

**26-** ECG repouso realizado por cardiologista HCPA: _______________________________________

**Antecedentes**

**27-** Hipertensão Arterial Sistêmica (1) Sim (2) Não

**28-** Tabagismo (1) Sim (2) Não

**29-** Dislipidemia (1) Sim (2) Não

**30-** Dor nas pernas quando caminha (1) Sim (2) Não

**31-** Quando pára de caminhar melhora (1) Sim (2) Não

**32-** Dor nas pernas à noite (1) Sim (2) Não

**33-** Formigamento em membros inferiores (1) Sim (2) Não

**34-** Tontura ao levantar subitamente (1) Sim (2) Não

**35-** Atividade física regular (1) Sim (2) Não

Cite as modalidades que pratica: ___________________________________________________

_________________________________________________________________________________

Quantas vezes na semana ( ) 1 ( ) 2 ( ) 3 ( ) 4 ( ) 5 ( ) mais vezes

Duração da atividade (minutos) ( ) – 20 ( ) 20 a 30 ( ) 30 a 45 ( ) 45 a 60 ( ) mais 60

Intensidade da atividade ( ) leve ( ) moderada ( ) intensa

**Medicações em uso**

Nome do medicamento: ______________________________________________________________

Dose: _____________________________________________________________________________

Nome do medicamento: ______________________________________________________________

Dose: _____________________________________________________________________________

Nome do medicamento: ______________________________________________________________

Dose: _____________________________________________________________________________

Nome do medicamento: ______________________________________________________________

Dose: _____________________________________________________________________________

Nome do medicamento: ______________________________________________________________

Dose: _____________________________________________________________________________

Nome do medicamento: ______________________________________________________________

Dose: _____________________________________________________________________________

**ANEXO IV: APROVAÇÃO DO PROJETO PELO COMITÊ DE ÉTICA DO HOSPITAL DE CLÍNICAS DE PORTO ALEGRE (HCPA)**
